# Congenital hypertrophy of retinal pigment epithelium (CHRPE) in patients with familial adenomatous polyposis (FAP); a polyposis registry experience

**DOI:** 10.1186/1756-0500-7-734

**Published:** 2014-10-18

**Authors:** Anwer Nusliha, Ushantha Dalpatadu, Binara Amarasinghe, Pramodh Chitral Chandrasinghe, Kemal Ismail Deen

**Affiliations:** North Colombo Teaching Hospital, Ragama, Sri Lanka; Faculty of Medicine, University of Kelaniya, Ragama, Sri Lanka

**Keywords:** Familial adenomatous polyposis coli, Retinal pigmentation, Ophthalmic screening

## Abstract

**Background:**

Familial Adenomatous Polyposis (FAP) is an autosomal dominant condition giving rise to multiple adenomatous polyps in the colon which invariably become malignant by the fourth decade. Congenital hypertrophy of retinal pigment epithelium (CHRPE) is one of its extra intestinal manifestations early in childhood seen, present in 90% of FAP population and is easy to detect.

**Findings:**

Patients diagnosed with FAP and at risk first degree family members were screened for CHRPE using a slit lamp and indirect ophthalmoscopy. The retina of 17 diagnosed FAP patients and 13 individuals at risk were examined. The site and size of CHRPE lesions were documented. Thirteen (76%) of 17 FAP patients (male-10, female – 7, median age - 30 years; range 15-55 years) had CHRPE lesions; seven (54%) had bilateral CHRPE lesions and six (46%) had unilateral lesions. A single lesion was detected in 6 (46%) while 7 (54%) patients had multiple lesions. Of 13 at risk individuals (7- male, female-6 ; median age 34; range 16-52 years), one was positive for CHRPE and 12 were free of retinal lesions. The sensitivity of the presence of a CHRPE lesion in association with colonic polyps in FAP was 76%, specificity 92%, positive predictive value 93%, and negative predictive value 75%.

**Conclusions:**

This study found a high sensitivity and specificity for a CHRPE lesion to be associated with colonic polyps of FAP and hence a useful screening method in a burdened health-care system. The method is minimally invasive and simple and would be of particular value in screening children at risk for FAP.

## Background

Familial adenomatous polyposis (FAP) is a genetic disorder transmitted in an autosomal dominant pattern [1, 2]. Patients develop multiple adenomatous polyps in the colon varying from a hundred to thousands in number. If left, these polyps will invariably transform, through the adenoma-carcinoma sequence, to colorectal malignancy [3–5]. Therefore early diagnosis and appropriate treatment is essential. Patients may develop extra intestinal manifestations such as congenital hypertrophy of retinal pigment epithelium (CHRPE), sebaceous cysts (51%), desmoids tumours (26%), and in lesser proportions, osteomas, lipomas (Gardner’s syndrome ) and extra intestinal malignancies; hepatomas, retinoblastomas and brain tumours (Turcot’s syndrome) [6–9]. FAP is also associated with the presence of polyps in the upper gastro intestinal tract in 90 to 100 percent [7, 8]. Familial adenomatous polyposis is caused by mutations in the adenomatous polyposis coli (APC) gene, a tumour suppressor gene, located in the long arm of chromosome 5 [10–12]. The condition is autosomal dominant making half of the off spring of an affected individual parent at risk of developing FAP [10, 11]. Disease has a hundred percent penetrance when the genotype is present. FAP related CHRPE may not be seen in all with the FAP genotype. For example, mutations in codon 1597 are known to be associated with desmoid tumours and absent CHRPE [13]. CHRPE was first described by Blair and Trempe in 1980 [8]. It is commonly caused by a truncating mutation in the codons between 463 and 1387 of the APC gene [14–19]. The global prevalence of CHRPE in individuals with the APC mutation is ninety percent [9]. This makes detection of CHRPE in asymptomatic individuals who are at risk an attractive screening option. It is the most common and earliest extra colonic manifestation amongst FAP populations which may be present at birth (80%) or shortly after birth [8, 15, 16]. It is a darkly pigmented lesion with a depigmented halo in the retina [17]. It may be single or multiple, unilateral or bilateral. The size of a CHRPE lesion is variable but most are similar in diameter to the optic disc [20]. Lesions may be oval, round or bean shaped. Oval CHRPE lesions with fishtail shaped hypo-pigmented change at one or both ends may be associated with FAP [21]. CHRPE has no malignant potential [22]. The prevelance of CHRPE in the normal population is between 1.2% to 4.4% [23] which increases its specificity for screening. The aim of our study was to assess the value of ophthalmic screening, to find the association of CHRPE lesions in individuals with FAP or their family members in a local population and to describe the pattern of distribution of CHRPE lesions in these individuals in order to assess the feasibility of using it as a low cost, non invasive screening test to detect FAP.

## Findings

### Material and method

All individuals with a diagnosis of familial adenomatous polyposis made at the professorial surgical unit, Ragama between 1996 and 2010, were traced using clinical notes, endoscopic registers and admission books. Proformas were prepared for FAP patients and relatives at risk. The proforma contained all details about the patient including a plan of follow up. All FAP patients and individuals at risk were invited for registration in the FAP registry. A consent form was prepared in three languages (Sinhala, English and Tamil) and informed written consent was taken from all individuals participating in the activities of the registry. Those who consented were subjected to ophthalmological screening.

Ophthalmological examination was performed by an experienced ophthalmologist at North Colombo Teaching Hospital, Ragama. In all individuals, the eyes were checked for visual acuity as screening, followed by dilatation of the pupils using tropicamide and phenylephrine ophthalmic solution. After the pupils were fully dilated, slit lamp and indirect ophthalmoscopic examinations were performed to identify CHRPE lesions. If found, the site, size, number and shape of lesions were documented. A common side effect of this procedure was transient blurring of vision, which reversed in a few hours. Those who had ophthalmological screening for CHRPE lesions underwent a flexible sigmoidoscopy to look for the presence of polyps in the colon. If they were found to have polyps in the colon, an upper gastro intestinal endoscopy was also performed to look for polyps in the stomach and duodenum which may have malignant potential. Data were analyzed using the Statistical Package for Social Science version 17.0 (SPSS 17.0, Chicago, Illinois, USA). The sensitivity, specificity, positive predictive value and negative predictive value of CHRPE lesions in FAP and those at risk were calculated. Ethical clearance was obtained from the National Research Council, Sri Lanka and the Ethics Review Committee of University of Kelaniya medical school at Ragama.

## Results

Seventeen diagnosed patients (male-10, female-7, median age- 30 years, range 15-55 years) with FAP and 13 individuals at risk (male-7, female-6, median age -34, range 16-52 years) were screened by an ophthalmologist. Of those with FAP, 13 (76%) were positive for CHRPE and 4 (24%) were negative. Six (46%) had a unilateral lesion and 7 (54%) had bilateral lesions. A solitary lesion in 6 (46%) and multiple lesions in the same eye were found in 7 (54%) patients. CHRPE lesions were either round or oval in shape. Of 13 individuals at risk, one had a single and large CHRPE lesion in one eye. Currently he does not have polyps in the colon and he is under surveillance for colonic and upper intestinal polyps. The remaining 12 were negative for both CHRPE in the retina and polyps in the colon. Response rate amongst affected individuals was 68% (17 out of 25 reponded) and the at risk population was 32% (13 out of 40). The sensitivity of CHRPE as a screening test for the presence of FAP is 76% (95% CI : 0.56 - 0.97) and the specificity is 92% (95% CI: 0.78 - 1.07). The positive predictive value is 93% (95% CI: 0.79 - 1.06) while the negative predictive value is 75% (95% CI: 0.54 - 0.96).

## Discussion

FAP is a genetic disorder which accounts for 1% of colorectal cancer [2, 3, 6]. Almost all patients develop colorectal cancer from adenomatous polyps unless detected early and managed by prophylactic removal of the colon and rectum. Therefore, early diagnosis is paramount. Currently the standard surgical treatment for FAP is restorative proctocolectomy with ileal pouch anal anastomosis. As CHRPE is one of the commonest and earliest extra intestinal manifestations, it lends itself as a screening tool for family members of FAP patients. The sensitivity of CHRPE in our study is 76% and it is comparable with other studies [17]. Chen CS et al reported 56% sensitivity in detecting FAP with CHRPE while none of the subjects with hereditary non polyposis colon cancer in their study had retinal lesions [18]. All retinal lesions present in FAP patients may not be CHRPE. These may be harmatomatous lesions which will be difficult to differentiate without special imaging techniques such as spectral-domain optical coherence tomography [24]. A drawback in our study was that ‘at risk’ individuals were assessed with endoscopy and not genetic studies. Some of these patients may have the genotype which is yet to manifest clinically. Although genetic testing is precise in most settings sigmoidoscopy is accepted as a screening tool due to financial constraints. We found that screening for CHRPE was relatively easy and caused minimum discomfort to the individual. Compared with colonoscopy, retinal screening seems more acceptable and less stigmatic. The examination is safe and repeatable. Although we did not study the cost benefit of retinal screening, it is our impression that retinal screening is less expensive compared with colonoscopy. Once CHRPE lesions are detected the screening of family members at risk could be intensified and should be done in a coordinated manner under the guidance of an FAP Registry. CHRPE detection can also help to persuade individuals at risk for regular follow up and improve their compliance. In Sri Lanka, financial constraints hinder genetic testing; screening for CHRPE will be most appropriate and invaluable to diagnose FAP. Furthermore, it may be suggested that CHRPE positivity will allow targeted genetic screening.

## Conclusions

Screening for CHRPE is an easy, simple to perform, non invasive, safe and reproducible method of diagnosing FAP. As shown in this study it can be used as an effective first-line screening method for FAP. In conjunction with other screening methods it is invaluable in early diagnosis of FAP patients. As patients’ compliance is good with this screening method we could screen many patients who are at risk for FAP enabling early and appropriate management for those with FAP.

## Abbreviations

CHRPE: Congenital hypertrophy of retinal pigment epithelium
FAP: Familial adenomatous polyposis coli.
